# An Extraordinary Case of Brain Surgery

**Published:** 1913-01-18

**Authors:** 


### AN EXTRAORDINARY CASE OF BRAIN SURGERY.

At the Section of Laryngology * on Friday, 10th inst., Mr. C. I. Graham brought forward a case (the first of its kind in this country) of cyst of the pituitary fossa, in which operation was performed by the nasal route. The patient was a woman at thirty-seven who was admitted to hospital on account of failure of sight, which had been progressive for eighteen months, temporal headaches, drowsiness, slow mental reaction, and several months' incontinence of urine. On admission the right eye was blind, there was optic atrophy, and the pupil showed only consensual light reflex. Vision was present in the left nasal field, the pupil was dilated, and showed neither direct nor consensual light reflex. Urine was normal, 16 to 24 oz. being passed daily; contained no trace of sugar twenty-four hours after giving 7½ oz. glucose. Skiagraphy showed the pituitary fossa to be flattened and enlarged. The temperature was 98.4° F., pulse 82, respirations 20. Eighteen days after admission the drowsiness had proceeded to coma, there was incontinence of both urine and fæces, the vision was worse, and respirations became slowed to nine or ten per minute. Operation was therefore performed by Mr. Graham on the following day under intravenous infusion of ether, and consisted of removal of most of the septal cartilage, vomer, and a small part of the vertical plate of the ethmoid, the incision splitting the columella and extending into the upper lip. The pituitary fossa was opened by Cushing's method, and about 2 drachms of fluid escaped from it. The superficial incision was closed by two stitches and dressed with collodion and wool. Before the return of consciousness the respirations were 24 per minute, the temperature 97° F., and the pulse 100. For twenty-four hours after operation thirst was intense, and there were frequency of micturition and polyuria—namely, 100 oz. The patient was excited and garrulous. Fifteen days after there had been no incontinence since the operation, and the patient, except for her sight, appeared to have become normal again. The President and several other members expressed their admiration of Mr. Graham's result and a desire for more detail in regard to procedure and symptoms.

At the meeting of the Clinical Section on Friday evening, 10th inst., Dr. J. D. Rolleston showed a boy at five years who had diphtheritic hemiplegia. He was admitted with very severe faucial and nasal diphtheria in the fourth day. 20,000 units of antitoxin were given on admission and two further doses of 24,000 units on each of the two following days. Cardiac dilatation and irregularity occurred on the sixth day and were accompanied by vomiting, continuing until the fifteenth day, and by enlargement of the liver. The knee jerks and ankle jerks were lost on the twenty-first day. Palatal palsy, shown by regurgitation of fluid through the nose, occurred on the twenty-ninth day, ciliary palsy on the twenty-third day, and pharyngeal palsy from the thirty-sixth to the fifty-eighth day. There occurred also paresis of rectal and vesical sphincters and of the diaphragm. There was albuminuria from the ninth to forty-first day, but there were no serum phenomena beyond a slight urticarial rash lasting one day. On the patient's discharge on the ninety-fourth day from the onset of the hemiplegia there was some spasticity of the right arm and leg, slight right facial palsy, extensor plantar response, ankle clonus, and combined movement of trunk and pelvis on right side. Intelligence was intact, but speech was limited to a few syllables.

The case was fully discussed and the general experience was that the prognosis of such a condition was unfavourable.

* Royal Society of Medicine.

